# Duration of Allergen Immunotherapy for Long-Term Efficacy in Allergic Rhinoconjunctivitis

**DOI:** 10.1007/s40521-018-0176-2

**Published:** 2018-08-31

**Authors:** Martin Penagos, Aarif O. Eifan, Stephen R. Durham, Guy W. Scadding

**Affiliations:** Allergy and Clinical Immunology, Division of Respiratory Science, National Heart and Lung Institute, Imperial College London, Royal Brompton Hospital Imperial College London, Dovehouse Street, London, SW3 6LY UK

**Keywords:** Allergic rhinitis, Allergen immunotherapy, Sublingual, Subcutaneous, Mechanisms, Biomarkers, Long term efficacy

## Abstract

**Rationale:**

Subcutaneous and sublingual immunotherapy are effective for allergic rhinitis. An important question is whether allergen immunotherapy provides a sustained clinical effect after treatment cessation. In view of potential side effects, cost and the necessary patient commitment, long-term benefit is an important consideration for the recommendation of immunotherapy over standard pharmacotherapy.

**Purpose of review:**

In this review, we analyse the existing evidence for long-term effects of both routes of administration in the context of double-blind, placebo-controlled, randomised clinical trials that included a follow-up phase of at least 1 year after treatment cessation.

**Recent findings:**

Overall, evidence suggests that 3 years of either subcutaneous or sublingual immunotherapy result in clinical benefit and immunological changes consistent with allergen-specific tolerance sustained for at least 2–3 years after treatment cessation.

**Summary:**

The data presented here support recommendations in international guidelines that both routes of administration should be continued for a minimum of 3 years. Gaps in the evidence remain regarding the long-term efficacy of immunotherapy for perennial rhinitis and studies performed in children.

## Introduction

Allergic rhinitis (AR) is the most common immunological disease [1]. It affects up to 30% of people in the USA [2], 10–15% of children and 26% of adults in the UK [3•, 4]; the overall prevalence in Europe is about 23% [4]. AR can cause bothersome symptoms, which may impair quality of life, productive time at work and school, sleep quality and reduce involvement in outdoor activities [2, 5]. Up to 40% of individuals with allergic rhinitis have or will go on to develop asthma [2, 6]. Standard treatment of allergic rhinitis consists of allergen avoidance and pharmacotherapy which includes the use of non-sedating oral antihistamines, topical intranasal antihistamines and intranasal corticosteroid sprays. Combinations of these are often needed, especially for moderate to severe forms of the disease [1, 2, 5]. When used appropriately, these medications are generally effective; nonetheless, these must be repeated when symptoms recur as the underlying allergic disease remains unmodified. Suboptimal responses are often observed due to poor treatment adherence or inability to tolerate these drugs [7–9]. Population surveys have reported that up to a third of children and up to two thirds of adults have partial or poor relief with pharmacotherapy alone [10, 11]. When subjects with AR have inadequate response to these antiallergic medications or have bothersome adverse effects, allergen immunotherapy should be considered [7, 8, 12–14].

Allergen immunotherapy administered subcutaneously (SCIT) has been the standard practice to treat AR whereas the sublingual route (SLIT) has emerged as an effective and safe alternative [7, 13–15]. Subcutaneous immunotherapy comprises the repeated administration of increasing concentrations of the relevant allergen weekly for 3–5 months followed by monthly maintenance injections [7, 16]. Patients on sublingual immunotherapy receive a fixed allergen dose once a day, which is administered continuously throughout the year or pre/co-seasonally, depending on the allergen that triggers the symptoms and the type of allergen extract used [3•, 7, 17, 18]. Maintenance doses for both subcutaneous and sublingual immunotherapy have traditionally been recommended to be continued for at least 3 years [2, 3•, 7, 8, 13, 14, 19]. Subcutaneous immunotherapy has been shown to be highly effective, especially for seasonal AR [16, 20], but also for perennial disease [21, 22]. Subcutaneous immunotherapy may occasionally be associated with allergic systemic effects such that it has to be administered by trained staff, in the presence of a physician, in a specialist setting with rapid access to adrenaline and other resuscitative measures [7, 23]. A number of well-powered double-blind, placebo-controlled randomised clinical trials (DBPCRCTs) have demonstrated that sublingual immunotherapy is an effective and safe alternative to the subcutaneous route for seasonal AR and for perennial disease in patients with house dust mite (HDM) allergy [7, 17, 18, 24, 25, 26•]. Local side effects are common in patients receiving SLIT [3•, 7, 17, 18, 24, 26•]. There have been isolated reports of more severe allergic side effects including anaphylaxis; however, there have been no fatalities [27].

An important question is whether allergen immunotherapy is able to induce clinical and immunological allergen-specific tolerance, which may be defined as the persistence of clinical benefits for at least 1-year after treatment discontinuation, accompanied by altered antigen-specific T-cell and/or B-cell responses [3•, 28, 29]. In this review, we analyse the available evidence on the long-term effects of subcutaneous and sublingual immunotherapy in the context of DBPCRCTs that included a follow-up phase of at least 1 year after treatment discontinuation. Three long-term DBPCRCTs of sublingual immunotherapy [9, 17, 18, 30–33], three of subcutaneous immunotherapy [34–38] and one of both sublingual and subcutaneous immunotherapy for allergic rhinitis [3•] are discussed. Additionally, a recent 1-year DBPCRCT of HDM SLIT with a further 1-year follow-up phase [26•] and two studies—one of subcutaneous [39], one of sublingual immunotherapy [40•]—for prevention of disease progression to asthma in children are also discussed.

## Long-term randomised controlled trials of subcutaneous immunotherapy for allergic rhinitis

Long-term treatment efficacy has been defined by the EAACI as sustained clinical benefit that lasts for at least 1 year after immunotherapy discontinuation and short-term treatment efficacy as the clinical benefit to the patient while they are receiving immunotherapy [13, 41]. A number of studies have aimed to assess these long-term clinical and immunological benefits of subcutaneous immunotherapy for allergic rhinitis after its discontinuation [13, 19, 41–43]. However, few studies have assessed efficacy for at least 12 months after cessation of immunotherapy in the context of randomised, double-blind, placebo-controlled clinical trials. One long-term DBPCRCT of subcutaneous grass pollen [34–36], one of ragweed [37] and one of *Parietaria* immunotherapy—albeit double-blind during the first year only— [38] were identified for inclusion in this review (Table 1).

**Table 1:** Long-term efficacy of subcutaneous immunotherapy. Randomised, double-blind, placebo-controlled clinical trials. Adapted with permission from [7]

Author, Year, Country	SCIT(n)	PLACEBO(n)	Patients characteristics	Allergen	Units	Cumulative dose	Total study duration (Years)	SCIT duration (Years)	Years after cessation	Years blinded after cessation	Dropout rate
Naclerio [37] 1997 USA	10	10	Age: 18-55 years old Dx: Ragweed induced hay fever Tests: ID skin test with ragweed extract. Asthma: Patients with mild asthma were included.	Ragweed	AU	480,000 AU ~1150 μg of *Amb a* 1	4-5	3-4	1	1	0% (Double-blind phase)
Durham [36], Walker [35], Varney [34] 1991, 1995, 1999 United Kingdom	21	19	Age: 19-52 years old Dx: Severe SAR associated with grass pollen Tests: Positive SPT to *P*. *pratense* Asthma: Patients with chronic asthma were excluded	*Phleum pratense*	SQ-U	~ 1400 μg of *Phl p 5*	7	Up to 7	3	3	Y1: 7.5% Y2: 17.5% Y3: 20%
Ariano [38] 1999 Italy	13	12	Age: 13-62 years old Dx: ARC with single sensitization to *Parietaria* Tests: Positive sIgE and SPT to *Parietaria* Asthma: 20% mild asthma	*Parietaria judaica and P. officinalis*	AU	Year 1: 100 000–120 000 AUeq Subsequent years: 120 000 AUeq	7	2-3	4	0	8%

*ARC* Allergic rhinoconjunctivitis, *SAR* Seasonal Allergic Rhinitis, *NA* Not available, *SQ-U* standardised quality units, *AU* Allergy Units, *SAR* Seasonal Allergic Rhinitis, *AIT* Allergen Immunotherapy, *ID* Intradermal, *NAC* Nasal allergen challenge, *NF* Nasal fluid, *SMS* Symptoms and medication scores, *Y* Year

In a 7-year trial with *Phleum pratense*, 40 adults with a history of severe SAR were initially randomised to receive subcutaneous immunotherapy (*n* = 21) or placebo (*n* = 19) over 1 year. Thirty-seven participants completed this phase. After this period, those on active treatment were invited to continue for a further 3 years, whilst those on placebo were invited to switch to active treatment for 3 years, resulting in 32 participants receiving either 3 or 4 years of immunotherapy. Thereafter, these remaining participants were randomised in a double-blind allocation to either continue receiving SCIT (maintenance group, *n* = 16) or to receive placebo injections (discontinuation group, *n* = 16) for the following 3 years. Fifteen matched grass-pollen allergic controls who had never received immunotherapy were monitored in parallel. The maintenance dose consisted of monthly injections of 20 μg of *Phl p* 5 (100,000 SQ-U). Placebo injections were identical vials of diluent, including aluminium and histamine. Notably, both symptom and medication scores remained low during the final 3 years, with no significant differences between participants who continued or discontinued immunotherapy. Total symptom scores in both immunotherapy groups (maintenance and discontinuation) were significantly lower than the matched control group (median area under the curve [AUC] 921, 504 and 2863, for maintenance, discontinuation and control groups, respectively) [34–36].

Ariano et al. conducted a randomised, placebo-controlled trial with subcutaneous *Parietaria judaica* and *Parietaria officinalis* immunotherapy, double-blinded during the first year. Active treatment consisted of a glutaraldehyde-modified allergoid [38]. Five hundred micrograms of the modified extract corresponded to approximately 20,000 AUeq (allergy units equivalent). The AIT schedule involved a build-up phase of increasing doses (from 1000 to 10,000 AUeq) which were administered weekly, followed by monthly maintenance injections. Twenty-five participants were enrolled (active *n* = 13, placebo *n* = 12). After completing 12 months of treatment, the active group continued SCIT for a further 2 years and the placebo group was switched to active treatment for 2 years. A subjective evaluation was conducted 4 years after completion of the full treatment period. The active group had significant reductions in symptom and medication scores (SMS) compared with placebo at 1 year of treatment (*p* = 0.02) (blinded phase). After switching to active SCIT, participants originally receiving placebo also improved, with reduced SMS compared to baseline in the following two seasons. Strikingly, when self-assessment questionnaires were completed a further 4 years after completion of the treatment period, both groups considered themselves to still be better than at baseline, with no difference between the group originally randomised to placebo treatment (and therefore having had only 2 years active treatment) and the group on active treatment from the start (therefore having received 3 years active treatment). Clearly, the format of this is less robust than the study by Durham et al., given the lack of blinding after the first year; however, the failure to show any difference between 2- and 3-year treatment is notable—suggesting perhaps that courses of treatment of less than 3 years could also have lasting effects, at least in the case of *Parietaria* immunotherapy [38].

Naclerio et al. recruited 20 adults who had received subcutaneous injections of 12 μg of *Amb a* 1 (5000 AU) fortnightly for at least 3 years [37]. Participants were randomised, blinded, either to continue on active treatment (*n* = 10) or to switch to placebo injections for 1 year (*n* = 10). Nasal allergen challenges (NACs) were performed before immunotherapy, at randomisation and 1 year after placebo-controlled treatment. After the initial 3-year open phase of immunotherapy, nasal challenges revealed decreases in the number of sneezes in all participants (median 7 to 1; *p* = 0.005). Analysis of nasal fluid showed reductions in TAME-esterase (*p* = 0.0004), histamine (*p* = 0.008) and kinins (*p* = 0.0004). After the final additional year of double-blind placebo-controlled treatment, the clinical and mediator response to NAC remained entirely suppressed in the group that remained on active treatment. Conversely, the group on placebo showed a partial recrudescence of response to NAC, with median number of sneezes increasing from 2 vs 4, and levels of nasal fluid TAME-esterase, histamine and kinins all increasing compared to levels seen after 3 years active treatment, albeit not to the same degree pre-immunotherapy levels. Of note, seasonal symptom scores were no different between those who remained on active treatment and those on placebo, although the authors point out that the study may have been underpowered to detect such a difference if it did exist. It should also be noted that ragweed-specific IgG antibodies declined following the switch to placebo injections but not in those who remained on active treatment.

Together, these three studies [34–38] suggest that a long-term tolerogenic effect of SCIT can be achieved following 3-year treatment, but that this effect is not absolute, and might differ depending on allergen used. Whilst the study of Durham et al [34–36] suggests no additional benefit in longer courses of treatment, the data on nasal fluid mediators and clinical response to nasal challenge provided in the study by Naclerio et al [37] does raise the possibility that the treatment effect may begin to diminish as early as 1-year off-treatment. However, how well this mediator data following nasal challenge relates to symptoms on usual seasonal exposure is unclear. More detailed mechanistic studies may allow clearer immunological–clinical correlates to be established.

## Long-term randomised controlled trials of sublingual immunotherapy for allergic rhinitis

**Fig. 1:**
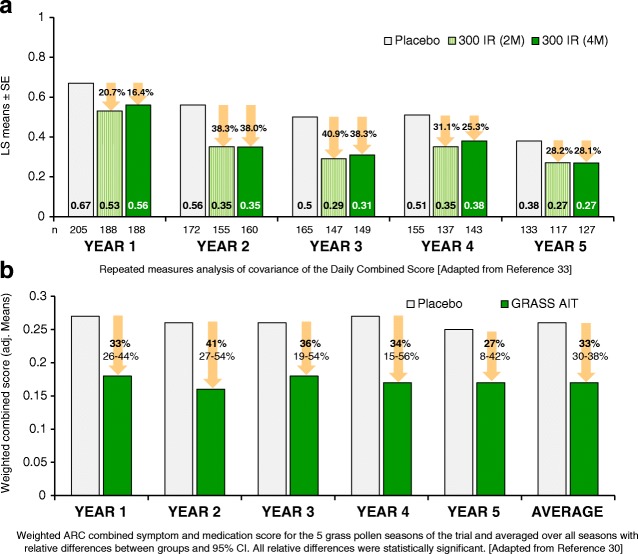
Effects of sublingual immunotherapy on combined symptom and medication scores in two 5-year, double-blind, placebo-controlled, randomised controlled trials. **a** Five-grass tablet [33]. **b** *Phleum pratense* tablet [30].

Three long-term DBPCRCTs of sublingual grass pollen immunotherapy for allergic rhinitis were identified for this review [9, 17, 18, 30–33], one using *Phleum pratense* tablets [9, 17, 30], one five-grass mix tablets [18, 32, 33] and one five-grass mix drops [31]. The long-term, posttreatment-discontinuation outcomes of these studies were broadly similar (Table 2 and Fig. 1).

**Table 2:** Long-term efficacy of sublingual immunotherapy. Randomised, double-blind, placebo-controlled clinical trials. Adapted with permission from [7]

Author, Year, Country	SLIT(n)	PLACEBO(n)	Patients characteristics	Allergen	Units	Cumulative dose	Total study duration (Years)	SLIT duration (Years)	Years after cessation	Years blinded after cessation	Dropout rate
Durham [9,30], Dahl [17] 2006, 2010, 2012 United Kingdom	316	318	Age: 18-65 years old Dx: 2-year history of grass-pollen induced ARC Tests: Positive sIgE and SPT to *P*. *pratense* Asthma: Patients with *perennial* asthma were excluded.	*Phleum pratense*	SQ-T	5.48 mg per 365-day period	5	3	2	2	Y1: 10.4% Y2: 50.2% Y3: 54.7% Y4: 59.5% Y5: 62%
Ott [31] 2009 Germany	142	67	Age: 7-64 years old Dx: ARC associated with grass pollen Tests: Positive sIgE and SPT to grass pollen Asthma: 11-14%	5 grasses mix	IR	22 000 IR per season (1500 μg of group 5 major allergen)	4	3	1	1	57.3% PP
Didier [18,32,33] 2011, 2013, 2015 France	207 (2M) 207 (4M)	219	Age: 18-50 years old Dx: 2-year history of grass-pollen induced ARC Tests: Positive sIgE and SPT to grass pollen Asthma: 11-16%	5 grasses mix	IR	9000 IR a month [750 μg group 5 major allergen a month]	5	3	2	2	Y1: 9.6% Y2: 23.2% Y3: 27.8% Y4: 31.6% Y5: 41.2%

*ARC* Allergic rhinoconjunctivitis, *SAR* Seasonal Allergic Rhinitis, *PP* per protocol, *SQ-T* standardised quality units tablets, *IR* Index of reactivity, *Y* Year, s*IgE* Specific-IgE

A 5-year randomised double-blind, placebo-controlled trial, consisting of a 3-year treatment phase followed by a further 2 years of blinded follow-up was conducted in adults with a history of moderate-to-severe grass pollen-induced allergic rhinoconjunctivitis [9, 17, 30]. Participants at randomisation were allocated to receive immunotherapy (*n* = 316) or matching placebo (*n* = 318). The treatment was started 16 weeks before the expected start of the first grass pollen season (year 1), with tablets taken daily for 3 years. Active treatment consisted of a fast-dissolving grass allergen tablet containing ~ 15 μg major allergen *Phl p 5* (75,000 SQ-T). Two hundred and thirty-eight subjects completed the 5-year follow-up. Mean symptom scores were reduced by 25 to 36% in the immunotherapy group compared with placebo over the five consecutive grass pollen seasons, including during 2 years off-treatment (*p* ≤ 0.004). Similarly, medication scores were reduced by 20 to 45% (*p* ≤ 0.022, seasons 1–4; *p* = 0.114, season 5), and the weighted combined SMS was reduced by 27 to 41% in the active group throughout the 5-year period (*p* ≤ 0.003). Efficacy was supported by improvements in quality of life, global symptom scores and the allergen-specific antibody response (significant increases in allergen-specific IgG4) in the active group. No serious safety issues in relation to treatment were reported. Nonetheless, local application site-related adverse reactions were described, the most common being oral and ear pruritus, mouth oedema and throat irritation [9, 17, 30].

A randomised, double-blind, placebo-controlled study evaluated the long-term efficacy and safety of a five-grass pollen mix of sublingual drops according to a co-seasonal schedule [31]. Adults and children with ARC were randomly allocated to receive either sublingual immunotherapy (*n* = 142) or placebo (*n* = 67) during three pollen seasons, followed by a further season of blinded follow-up. SLIT consisted of a mixture of pollen extracts of five grasses (cocksfoot or orchard, meadow, perennial rye, sweet vernal and timothy) at a concentration of 300 IR/mL (equivalent to 21 μg/ml of *Phl p* 5). A 1-day titration was performed at the estimated start of each pollen season (30–300 IR), followed by 300 IR daily for the duration of each season. The mean treatment duration ranged from 82 to 93 days across the four seasons. By the third season, median SMS had decreased by 45% in the active and 15% in the placebo group compared with baseline values, with significant differences between groups each year (*p* = 0.043, *p* = 0.040 and *p* = 0.0019, years 1, 2 and 3, respectively). Scores were still lower in the actively treated group 1 year after discontinuation (year 4), but statistical significance was narrowly missed (*p* = 0.052). Symptom scores alone, however, were significantly reduced at year 4 in the active-discontinued group versus placebo (*p* = 0.015). The levels of allergen-specific IgG_4_ increased significantly in the active group (*p* < 0.005), but no differences were observed compared with placebo after immunotherapy withdrawal. No serious systemic or anaphylactic reactions were observed [31].

The long-term efficacy of a five-grass pollen sublingual tablet was assessed in adults with grass pollen-induced ARC [18, 32, 33]. Six hundred and thirty-three adults were randomised to receive placebo (*n* = 219) or sublingual immunotherapy 2 (2M) or 4 (4M) months before the expected start of the pollen season (*n* = 207 and 207, respectively). Treatment was then continued daily throughout the season for three consecutive years. Study years 4 and 5 were off-treatment, blinded follow-up. Three hundred and seventy-seven participants completed the 5-year follow-up (placebo = 133, 2M = 117 and 4M = 127). Least squares (LS) for the mean daily combined score (DCS) was reduced by 16 to 38% in the 4M group compared with placebo during the five pollen seasons covered by the trial. The daily ARC total symptom score (DRTSS) was reduced by 11 to 39% and the daily rescue medication score (DRMS) reduced by 23 to 38% in the 4M group compared with placebo. During the first and second off-treatment years, a statistically significant difference was observed in LS mean daily combined score in the 4M group compared with placebo (25%, *p* = 0.0103, and 28%, *p* = 0.0478, respectively) [ 18, 32, 33].

These three studies provide robust evidence for induction of lasting tolerance after 3 years of grass pollen SLIT [9, 17, 18, 30–33]. The failure of the study by Ott et al [31] to reach statistical significance of the primary outcome—combined SMS—1 year after treatment completion (and the failure to maintain elevated allergen specific IgG4 levels) might relate to the shorter treatment periods (and therefore lower overall dose), than the other two studies. This is also consistent with the greater treatment effect seen in the group receiving 4 months compared to those receiving 2 months pre-seasonal treatment in the study by Didier et al [18, 32, 33].

In addition to the above studies of grass pollen SLIT, a recent study of HDM sublingual immunotherapy evaluated the persistence of effect 1 year after completion of treatment [26•]. Five hundred and nine adults with HDM-associated allergic rhinitis were randomised to receive placebo (*n* = 170) or HDM SLIT tablets at doses of 500 IR (*n* = 169) or 300 IR (*n* = 170) daily for 1 year [26•]. Eighty-four percent of participants completed the first year and 78% the treatment-free follow-up period. After 12-month treatment, the average adjusted symptom scores were reduced by 20.2% in the 500 IR group (*p* = 0.0066) and by 17.9% in the 300 IR group (*p* = 0.015) compared with placebo. The effect was maintained during the subsequent immunotherapy-free year (19.1 and 17%, respectively). Adverse events were mainly local (oral pruritus, throat irritation and mouth oedema) and were generally mild to moderate in intensity. No cases of anaphylaxis or epinephrine use were reported [26•]. This sustained clinical benefit after just 1 year of treatment raises the question of whether shorter courses of HDM sublingual immunotherapy may be sufficient for tolerance, possibly related to continuous environmental exposure to house dust mites, in contrast to the seasonal exposure with pollen allergens. Nonetheless, confirmation in a more prolonged study is needed to establish the duration of the treatment effect after mite immunotherapy.

The long-term effects of immunotherapy have been also evaluated in the context of open, non-blinded studies of up to 12–15 years [44, 45]. Although these and similar studies have shown clinical benefit of immunotherapy throughout diverse immunotherapy-free follow-up periods and are generally supportive of long-term benefits after discontinuation, the lack of a blinded placebo control group limits the weight given to these findings.

## Long-term randomised controlled trials of sublingual and subcutaneous immunotherapy for allergic rhinitis

A three-parallel-group DBPCRCT was conducted to evaluate whether 2 years of grass pollen sublingual immunotherapy is able to induce persistent effects 1 year after treatment discontinuation in patients with moderate to severe seasonal allergic rhinitis (Table 3**)** [3•]. One hundred and six adults were randomised to receive either 2 years daily sublingual immunotherapy with a *Phleum pratense* tablet (15 μg *Phl p* 5) plus monthly placebo injections (*n* = 36) or monthly *Phleum pratense* subcutaneous immunotherapy (20 μg *Phl p* 5) plus daily placebo tablets (*n* = 36) or double-placebo (*n* = 34), followed by a further 1-year follow-up off-treatment. Nasal allergen challenges were performed at baseline and at 1, 2 and 3 years. Ninety-two participants completed the 3 years (87%). At year 2 (on treatment), both treatments were highly effective at suppressing nasal allergen challenge with a 42% (*p* < 0.01) and 27% (*p* < 0.02) reduction in the total nasal symptom score for subcutaneous and sublingual immunotherapy, respectively. There were corresponding reductions in peak nasal inspiratory peak flow of 54% (*p* < 0.01) and 45% (*p* = 0.01), respectively. The trial was not powered to detect differences between active treatments. Nonetheless, subcutaneous immunotherapy was more effective than sublingual immunotherapy in reducing symptoms after nasal allergen challenge at year 1.

**Table 3:** Long-term efficacy of sublingual and subcutaneous immunotherapy. Randomised, double-blind, placebo-controlled clinical trials

Author, Year, Country	SCIT (n)	SLIT (n)	PLACEBO (n)	Patients characteristics	Allergen	Units	Cumulative dose	Total study duration (Years)	Immunotherapy duration (Years)	Years after cessation	Years blinded after cessation	Dropout rate
Scadding [3•] 2017 United Kingdom	36	36	34	Age: 18 to 65 years old Dx: Moderate to severe ARC Tests for DX: SPT, sIgE, NAC Asthma: Mild asthma	*Phleum pratense*	SQ-T SQ-U	~11 mg Phl p 5 SLIT ~ 0.55 mg Phl p 5 SCIT	3	2	1	1	13%

*NAC* Nasal allergen challenge

One year after completing treatment, allergen-induced total nasal symptom scores (TNSS) in the sublingual and subcutaneous immunotherapy groups did not significantly differ from placebo (*p* = 0.75 and 0.052, respectively). In contrast, both forms of immunotherapy had significantly smaller early (SLIT *p* < 0.003; SCIT *p* < 0.001) and late (SLIT and SCIT *p* < 0.001) skin responses compared to placebo at this stage. Adverse reactions to sublingual immunotherapy were generally mild, transient, local oral or upper gastrointestinal symptoms. Subcutaneous immunotherapy was associated with the expected rate of systemic reactions, and adrenaline was administered to two participants who presented with grade III reactions.

This study demonstrated that 2-year treatment was not sufficient for sublingual immunotherapy to achieve an improvement in the allergic response 1 year after treatment discontinuation, as judged by response to nasal allergen challenge. Similarly, 2 years of subcutaneous immunotherapy also just failed to produce clinical tolerance after 1 year off-treatment (*p* = 0.052) [3•].

## Long-term randomised controlled trials of subcutaneous and sublingual immunotherapy for prevention of progression of rhinitis to asthma

Long-term prevention has been defined by the EAACI as the protective effect of allergen immunotherapy against the development of new sensitizations or new allergic disease which is maintained for two or more years after treatment is completed [46, 47]. In a large clinical trial, 205 children aged 6 to 14 with moderate to severe rhinoconjunctivitis due to grass and/or birch pollen were randomised either to receive subcutaneous immunotherapy (*n* = 102) or to an open control group (*n* = 103). All subjects had hay fever symptoms, but none had persistent asthma symptoms at inclusion. Allergen immunotherapy included a weekly build-up phase followed by maintenance injections [20 μg of Phl p 5 (grass) or 12 μg of Bet v 1 (birch)] which were given every 6 weeks (± 2 weeks) for 3 years. Participants in the active group had lower risk of developing asthma 0, 2 and 7 years after subcutaneous immunotherapy discontinuation [OR 95%CI 2.52 (1.3–5.1), 2.68 (1.3–5.7), 4.6 (1.5–13.7), respectively] [39].

Recently, a large, double-blind, placebo-controlled trial that included 812 children aged 5–12 was conducted to determine the long-term effects of a grass pollen sublingual tablet immunotherapy and the risk of developing asthma [40•]. Participants with grass pollen allergic rhinoconjunctivitis were randomised to receive sublingual immunotherapy (*n* = 398) or placebo (*n* = 414) during 3 years, and then, they were followed up for 2 years after treatment cessation. Tablets contained 15 μg major allergen *Phl p* 5 (75,000 SQ-T). The primary endpoint was time to onset of asthma, measured in days from randomisation. After 5 years, there was no difference in time to onset of asthma (*p* = 0.667), defined by documented reversible impairment of lung function. However, participants in the sublingual immunotherapy group had a reduced risk of experiencing asthma symptoms or using asthma medication [OR (95%CI) = 0.66 (0.45–0.97), *p* < 0.036]. The study was powered on an anticipated 20% onset of asthma within 5 years for the placebo group. One explanation for the lack of effect on the primary endpoint was the very low prevalence of pre-defined asthma (i.e. with reversible impairment of lung function) for either group (9% in the placebo group). In addition to achieving the secondary endpoint of reduced asthma symptoms and asthma medication, the study confirmed the long-term effects of sublingual grass tablet immunotherapy on rhinoconjunctivitis symptoms in children. More evidence regarding the effect of immunotherapy in preventing new allergic sensitisations, onset of first allergic disease, or in the prevention of allergic comorbidities is needed [46, 47].

## Discussion

Subcutaneous and sublingual immunotherapies for respiratory allergy are highly effective and represent the most widely prescribed routes of administration [7, 12–15]. An essential question is whether allergen immunotherapy provides a sustained clinical effect after treatment cessation. Lack of this prolonged effect would question the viability of immunotherapy as an alternative to standard pharmacotherapy due to cost, potential side effects and the time commitment and inconvenience involved for patients [15, 19, 36].

In this review, we report the findings of randomised controlled trials that compare the long-term effects of subcutaneous or sublingual immunotherapy with placebo. The studies on subcutaneous immunotherapy included small samples of participants per group (*n* = 10–21) [34–38]. Nevertheless, in one study, it has been shown that 3 to 4 years of treatment result in persistent improvement in symptoms and reductions in rescue medication use at 3 years following double-blind withdrawal [34–36]. Further studies with similar design might help identify key immunological correlates of clinical tolerance and distinguish these from more transient bystander effects. The two other subcutaneous immunotherapy studies discussed here provide some discrepancies in their results. The study of *Parietaria* subcutaneous immunotherapy showed the treatment to be effective over 1 year versus placebo in a double-blind setting; the rest of the study, however, was unblinded [38]. The relevant finding for our purposes is that efficacy appeared to be maintained at 4 years after completion of treatment, irrespective of whether participants had received 2 or 3 years treatment. Conversely, in the study of ragweed subcutaneous immunotherapy, the effects of treatment for 3 years were already beginning to wane at 12 months off-treatment, albeit in the context of nasal allergen challenge outcomes [37]. These discrepancies might be explained by differences in the allergens used, but more likely by the different study designs—we are inclined to lend more weight to the outcome of the latter study in this regard. Two studies of grass pollen allergen immunotherapy tablets administered daily either pre-co-seasonally [18, 32, 33] or continuously [9, 17, 30] for 3 years gave similar results. In both studies, there was an approximate 30–40% reduction in symptoms and rescue medication during 3-year therapy and 20–30% reduction during 2 years off-treatment when double-blinding was maintained. Three years of sublingual drops of a five-grass pollen extract also still had a beneficial effect 1 year after discontinuation [31]. Local side effects were common but in general well-tolerated, and there were no serious adverse events reported. The question of whether perennial treatment over 3 years, as opposed to pre-co-seasonal treatment, induces a more robust (and perhaps longer lasting) clinical tolerance requires further clarification. The study by Scadding et al. [3•] demonstrated that 2 years of continuous sublingual immunotherapy did not provide significant long-term efficacy after 1 year of treatment discontinuation using nasal challenge response as the primary outcome. On the other hand, participants receiving either sublingual or subcutaneous immunotherapy had significantly reduced early and late skin responses (secondary outcomes) than placebo after 1 year off-treatment [3•]. It may be that 2-year treatment is enough to induce some lasting immunological changes, but that these are insufficient to maintain clinical improvement. Additionally, the outcome measures of this study—in the context of nasal allergen challenge—differ from the symptom-medication records during seasonal exposure of the other grass pollen studies discussed above. Nasal challenge may overwhelm any remaining protective effect in a manner that might not occur during usual seasonal allergen exposure.

## Conclusion

Taken together, these trials show that immunotherapy given for periods shorter than 3 years may be associated with relapse of symptoms after 1 year of treatment cessation, in contrast with studies in which treatment was given for at least 3 years [3•, 30, 31, 33, 36]. The finding that just 1 year of HDM SLIT provided clinical benefit a year after completion of treatment is a notable exception to this [26•]. Further confirmation and longer term studies are required. Additional areas for consideration include the role of nasal allergen challenge and environmental challenge chambers in assessing both short- and long-term effects of allergen immunotherapy. These direct challenge techniques have the advantages of providing precise, consistent levels of allergen exposure and generally do not require such large numbers of participants as natural exposure studies.

For now, clinicians should therefore be advised to follow established guidelines that recommend at least 3 years of allergen immunotherapy in order to achieve disease modification and long-term clinical and immunological tolerance [3•, 12, 13, 48, 49•].

## Abbreviations

AR: Allergic rhinitis
ARC: Allergic rhinoconjunctivitis
AIT: Allergen immunotherapy
SLIT: Sublingual immunotherapy
SCIT: Subcutaneous immunotherapy
DBPCRCT: Double-blind, placebo-controlled, randomised clinical trial
SPT: Skin prick test
HDM: House dust mites
AUC: Area under the curve
OR: Odds ratio
EAACI: European Academy of Allergy and Clinical Immunology
